# Bioequivalence of China- and Germany-manufactured bisoprolol (Concor^®^) tablets in fasted and fed healthy Chinese participants: a randomized, open-label, two-way, crossover study

**DOI:** 10.3389/fphar.2025.1527623

**Published:** 2025-03-12

**Authors:** Hang Yin, Zhili Jin, Ran Li, Dandan Li, Lusha Wang, Ruihua Dong

**Affiliations:** ^1^ Research Ward, Beijing Friendship Hospital, Capital Medical University, Beijing, China; ^2^ Merck Serono (Beijing) Pharmaceutical R&D Co., Ltd., Beijing, China

**Keywords:** bioequivalence, bisoprolol, clinical trial, pharmacokinetic, safety

## Abstract

**Background:**

The bioequivalence of Concor^®^ (Merck Healthcare KGaA, Darmstadt, Germany), a bisoprolol-containing tablet, manufactured in China and Concor^®^ tablets manufactured in Germany has not been previously reported.

**Methods:**

This single-center, open-label, randomized, two-period, two-sequence, crossover trial (28 February 2023–19 May 2023) compared the pharmacokinetics and safety of bisoprolol 5-mg tablets manufactured in China (test product) with those of bisoprolol 5-mg tablets manufactured in Germany (reference product) in healthy Chinese adults under fasted and fed conditions. Primary endpoints were C_max_, AUC_0–tlast_, and AUC_0– ∞ _.

**Result:**

The mean (coefficient of variation percentage) C_max_ in the fasted group was 21.2 (15.0) ng/mL (test product) and 22.1 (17.0) ng/mL (reference product). Under fed conditions, the respective C_max_ values were 22.7 (18.8) ng/mL and 22.8 (15.2) ng/mL. The mean and coefficient of variation percentage for AUC were also similar between the two products. The geometric least squares mean ratio (90% confidence interval) for the test/reference product was 0.9565 (0.9006–1.0158) ng/mL for C_max_, 0.9761 (0.9370–1.0168) h·ng/mL for AUC_0–tlast_, and 0.9807 (0.9429–1.0200) h·ng/mL for AUC_0– ∞_ in fasted conditions and 0.9966 (0.9289–1.0691) ng/mL for C_max_, 0.9672 (0.9220–1.0145) h·ng/mL for AUC_0–tlast_, and 0.9693 (0.9253–1.0155) h·ng/mL for AUC_0– ∞_ in fed conditions, which met the pre-defined criteria for bioequivalence. No serious treatment-emergent adverse events or deaths were observed.

**Conclusion:**

This study compared the bioequivalence of bisoprolol 5-mg tablets manufactured in China to that of the tablets manufactured in Germany among healthy Chinese adults.

**Systematic Review Registration:**

identifier CTR20230391

## 1 Introduction

Hypertension is a leading treatable cause of premature death (Zhou et al., 2021; Nguyen and Chow, 2021). In 2019, an estimated 1.3 billion adults were affected by hypertension, with the disease contributing to over 10 million deaths each year (World Health Organization, 2023). Hypertension is an important risk factor for cardiovascular disease (Fuchs and Whelton, 2020), including heart failure and coronary artery disease. The global prevalence of heart failure is increasing, which is thought to be the result of an aging population (Savarese et al., 2023), and coronary artery disease is the leading cause of death worldwide (Ralapanawa and Sivakanesan, 2021). Thus, the overall goal when treating patients with hypertension is to reduce the risk of cardiovascular disease.

The 2018 European Society of Cardiology/European Society of Hypertension guidelines consider β-blockers useful for treating hypertension, including in patients with symptomatic angina, post-myocardial infarction, and heart failure with reduced ejection fraction (Williams et al., 2018). Concor^®^ (Merck Healthcare KGaA, Darmstadt, Germany) tablets contain bisoprolol, a highly potent, synthetic, β_1_-selective adrenoceptor blocking agent. Bisoprolol has an absolute bioavailability of approximately 90% after oral administration. Bisoprolol is removed from the organism via two equally effective clearance routes: 50% is transformed into inactive metabolites in the liver with the excretion of the metabolites via the kidneys. The remaining 50% is excreted as unchanged substance via the kidneys. Bisoprolol reaches the maximal effect 3–4 h after oral administration. The plasma elimination half-life of 10–12 h provides 24-h efficacy following a once daily dosing (Merck, 2014). Bisoprolol exerts its therapeutic effects by blocking β receptors in the heart, resulting in a decrease in the myocardial contractility and a reduction in the blood pressure (Eguchi, 2016; Tjandrawinata et al., 2012).

Concor^®^ tablets are currently manufactured in Germany, and a newer manufacturing location has recently opened in China. In China, regulatory filing requires evidence for the bioequivalence of drugs manufactured domestically to reference drugs manufactured abroad (in Germany). To meet these requirements, we aimed to evaluate the bioequivalence of Concor^®^ 5-mg tablets manufactured in China and of Concor^®^ 5-mg tablets manufactured in Germany in healthy Chinese adults under both fasted and fed conditions. The secondary objectives were to assess the pharmacokinetic (PK) profiles, safety, and tolerability after a single dose of each of the tablets.

## 2 Patients and methods

### 2.1 Study design and treatments

This phase 1, single-center, open-label, randomized, two-period, two-sequence, crossover trial conducted in China between 28th February 2023 and 19th May 2023 compared the PK and safety of bisoprolol 5-mg tablets manufactured in China with those of bisoprolol 5-mg tablets manufactured in Germany. The study duration was approximately 4 weeks and consisted of a 2-week screening period, a 3-day dosing/sampling period, a washout period of at least 7 days, and a 3-day second dosing/sampling period, and the study ended on day 10 of the second dosing period.

**FIGURE 1:**
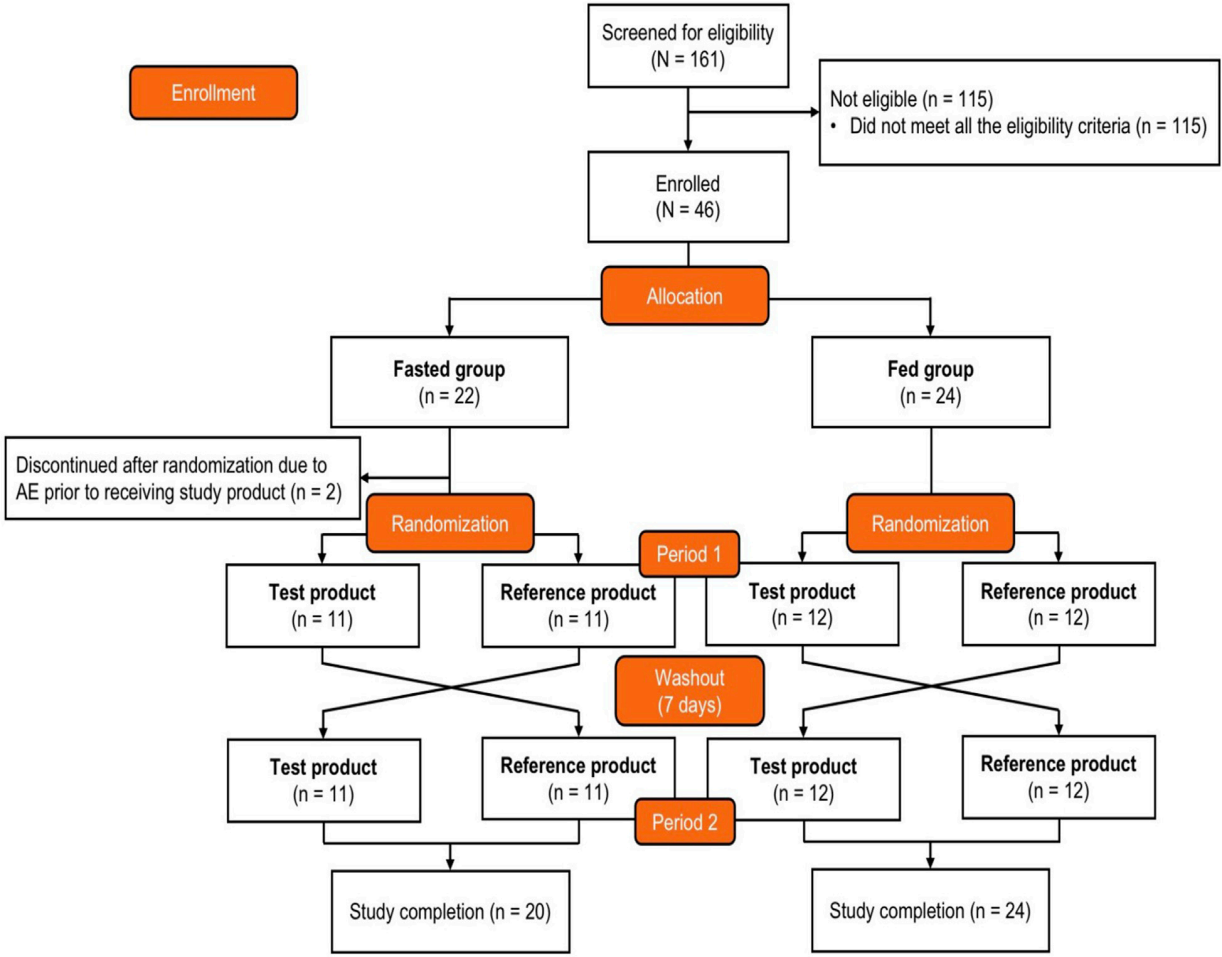
Study design and participant disposition. After enrollment and allocation to the fasted group or fed group, participants were randomized (1:1) to receive either the test product first and then the reference product second or the reverse order. The test product was bisoprolol 5-mg tablets manufactured in China, and the reference product was bisoprolol 5-mg tablets manufactured in Germany. AE, adverse event.

Healthy participants were allocated into either the fasted or the fed group, with each sex representing at least one-quarter of the participants in each group, and assigned a computer-generated randomization number. Allocated participants were then randomly assigned in a 1:1 ratio to Sequence A or Sequence B. Participants in Sequence A received the test product {one bisoprolol 5-mg tablet manufactured in China [Merck Pharmaceutical Manufacturing (Jiangsu) Co., Ltd., Nantong, China]} first and the reference product [one bisoprolol 5-mg tablet manufactured in Germany (Merck Healthcare KGaA, Darmstadt, Germany)] next, and those in Sequence B received the reference product first and the test product next (Figure 1).

The fasted group refrained from all food and drink, except water, from the evening (after dinner) of the day before the intervention for a total fasting time of at least 10 h. Water was allowed until 1 h before receiving the intervention, and then it was allowed again from 2 h after the intervention. The fed group consumed a standard high-fat, high-calorie breakfast, as per the United States Food and Drug Administration guidelines (U.S. Food and Drug Administration, 2002), within 30 min prior to receiving the intervention.

The study was conducted in compliance with the ethical principles outlined in the Declaration of Helsinki, the International Council on Harmonization Guidelines for Good Clinical Practice Council, and all applicable local laws and regulations. The study protocol and other relevant documents were approved by the Beijing Friendship Hospital Institutional Review Board and Ethics Committee (approval number: 2022-P1-drug-071-01). Participants provided written informed consent, and the clinical trial was registered in the Chinese Clinical Trials Register under the identifier CTR20230391.

### 2.2 Participants

Eligible participants were aged ≥18 years, considered healthy by medical evaluation (including medical history), had a body weight between 50 and 90 kg, and had a body mass index (BMI) between 19 and 26 kg/m^2^ (inclusive). The key exclusion criteria were positive screening for hepatitis B-soluble antigens or hepatitis C antibodies, human immunodeficiency virus, or *Treponema pallidum* antibodies; abnormal chest x-ray or computed tomography finding at screening; blood donation ≥400 mL or significant blood loss within 90 days prior to the administration of the first intervention; non-acceptance of the study’s high-fat breakfast; receipt of prescription or non-prescription medication within 28 days before the administration of the first intervention (including multivitamins and herbal products); participation in another clinical trial within 90 days prior to the first intervention administration; and any condition that was considered an inappropriate risk or contraindication for participation in the study.

### 2.3 Study assessments

The primary endpoints were the maximum observed concentration (C_max_), the area under the plasma concentration–time curve (AUC) from time 0 to the time of the last measurable concentration (AUC_0–tlast_), and the AUC from time 0 to infinity (AUC_0– ∞ _). PK parameters were evaluated using 3-mL blood samples that were collected via an indwelling cannula or direct venipuncture prior to the intervention and at 0.5, 1, 1.5, 2, 3, 4, 6, 8, 10, 12, 15, 24, 30, 36, and 48 h after the intervention. Blood samples were centrifuged at 4°C for 10 min at 2,000 g to separate the plasma supernatant, and the plasma was divided into two aliquots and stored at −20°C or lower.

The secondary endpoints included the time of the maximum observed concentration (t_max_), elimination half-life (t_1/2_), AUC from the time of the last quantifiable concentration extrapolated to infinity as a percentage (%AUC_extra_), apparent terminal elimination rate constant (λ_z_), apparent plasma clearance of the drug following extravascular administration (CL/F), and apparent volume of distribution during the terminal phase following extravascular administration (V_z_/F).

Baseline characteristics included weight, height, BMI, body surface area, alcohol consumption, nicotine use, drug screening, ethanol test, and viral serology. The dissolution profiles of the two products were determined using high-performance liquid chromatography, and the peak areas in the chromatogram were identified using the external standard method. The dissolution medium was sodium chloride buffer prepared in accordance with the European Pharmacopeia recommendations on dissolution testing (Ph. Eur. 5.17.1.), adjusted to pH 1.2, pH 4.5, and pH 6.8. Samples were evaluated using ultraviolet spectroscopic analysis at 5, 10, 15, 30, and 45 min.

### 2.4 Analytical method and method validation

Plasma concentrations of bisoprolol were analyzed using a fully validated liquid chromatography–tandem mass spectrometry (LC–MS/MS) method at Labcorp (Shanghai, China). Briefly, bisoprolol and its deuterium-labeled internal standard (bisoprolol-d5) were isolated from plasma (50 μL) using a 96-well plate following a protein precipitation extraction procedure with acetonitrile. The chromatographic separation was achieved using an ACQIUITY UPLC BEH C18 column (2.1 mm × 50 mm, 1.7 μm particle size; Waters Corporation) at a flow rate of 0.5 mL/min with a 3-min gradient profile, where the HPLC run began with the mobile phase B at 40% for the first 0.1 min, then increased to 95% at 1.5 min, held at 95% B until 2 min, and then returned to the starting composition at 2.1 min for column re-equilibration. Mobile phase A was 20 mM ammonium acetate, and mobile phase B was methanol, both containing 0.1% formic acid. The analytes were detected using a triple–quadruple mass detector (Sciex API 5500) in the positive ion mode, with turbo ion spray in multiple-reaction monitoring (m/z 326.2 → 116.2 for bisoprolol and m/z 331.3 → 121.1 for bisoprolol-d5). The method showed good linearity in the range of 0.500–75 ng/mL, with a lower limit of quantification set at 0.5 ng/mL. The intra- and inter-assay precision values (≤4.4% and ≤4.8%, respectively) met the acceptance criteria as per the regulatory guidelines. A battery of stability studies (i.e., bench-top, freeze–thaw, and long-term stability) were performed, and bisoprolol was stable in plasma for 24 h at room temperature and for 135 days at −30∼−10°C after five freeze/thaw cycles. No matrix effect was observed within the linearity range. The assay method was found to be highly reproducible and robust.

### 2.5 Safety assessments

The type, frequency, and severity of adverse events (AEs), including treatment-emergent AEs (TEAEs), and the relationship of AEs to the study intervention were recorded. AEs were coded using the Medical Dictionary for Regulatory Activities version 25.1, and TEAEs were assigned to a system organ class and preferred term. The additional safety assessments included vital signs (systolic and diastolic blood pressure, pulse rate, body temperature, and respiration rate), clinical laboratory tests (biochemistry, hematology, and urinalysis), 12-lead electrocardiogram results, and physical examinations.

### 2.6 Statistical methods

The sample size was determined from the bisoprolol intra-individual variability observed in a previous Merck bioequivalence study (EMR200006-001) that compared a fixed-dose-combination 5 mg/5 mg bisoprolol–amlodipine tablet with a 5-mg bisoprolol tablet co-administered with a 5-mg amlodipine tablet under fasted and fed conditions (Hu et al., 2018). In that study, the intra-individual coefficients of bisoprolol for AUC_0–tlast_, AUC_0– ∞ _, and C_max_ were, respectively, 10.4%, 10.6%, and 9.4% under fasted conditions and 13.5%, 13.4%, and 15.0% under fed conditions. Thus, considering the bioequivalence criteria for AUC_0–tlast_, AUC_0– ∞ _, and C_max_ (0.80–1.25) and allowing the test/reference ratio to vary between 0.95 and 1.05, a total of 16 participants in the fasted group and 18 in the fed group were considered sufficient to provide 95% power to confirm bioequivalence in each group and 90% power to confirm bioequivalence in both groups. Accounting for an attrition rate of 25%, a total sample size of 46 participants was planned, with 22 participants in the fasted group and 24 participants in the fed group.

The full analysis set (FAS) included all randomized participants. The safety analysis set (SAF) included all participants who had received at least one dose of the study product. The PK parameter analysis set (PKPS) included all participants who had received at least one dose of the study product and had no clinically important protocol deviations or events that would affect the PK analysis. The bioequivalence analysis set (BES) included all participants who had at least one evaluable PK parameter in at least one treatment period and who did not have any relevant protocol deviations, had no factors likely to affect the comparability of PK results, and had adequate study intervention compliance.

Continuous variables were summarized using descriptive statistics [including the number, arithmetic mean, median, standard deviation (SD), and minimum and maximum values], and quantitative variables were summarized by numbers and percentages. Geometric least-squares (LS) mean, coefficient of variation (CV), intra-participant CV percentage, and confidence intervals (CIs) were also calculated. No statistical comparisons were performed on the participant demographics or baseline characteristics.

Analysis of the primary endpoints was conducted in the BES. The data were log-transformed, and a mixed-effects model was applied, including fixed effects for sequence, treatment, and period. Differences between treatments on the log scale were estimated for the parameters, together with their 90% CIs. The geometric LS means and their 95% CIs were also estimated. The bioequivalence of the two tablets was assessed separately for the fasted and fed groups, and bioequivalence was only established if the 90% CIs for the geometric mean ratios for both primary endpoints between the two interventions were within 0.80–1.25 in both the fasted and fed groups. For t_max_, Hodges–Lehmann estimates and the corresponding 90% CIs were calculated according to the Tukey method. The 90% CIs of the geometric mean of the test product and reference product were calculated for all other secondary endpoints, but no formal statistical comparisons were performed. The dissolution profiles were characterized by plotting the dissolution rate over time, and the profiles of the two tablets were compared using a model-independent approach (U.S. Food and Drug Administration, 1997).

TEAEs with missing data regarding the association with the study intervention were considered related to the study intervention. No other imputation for missing values was conducted, and outliers were defined as values outside 1.5 × the interquartile range above the upper quartile or below the lower quartile.

PK parameters were derived using Phoenix^®^ WinNonlin^®^ version 8.3 (Certara, L.P., Princeton, NJ, United States), and all other statistical analyses were conducted using SAS^®^ version 9.4 or higher (SAS Institute, Cary, NC, United States).

## 3 Results

### 3.1 Participants

The participant disposition is shown in Figure 1. A total of 161 adults were screened, and 46 eligible participants were allocated to the fasted group (n = 22) or the fed group (n = 24) and randomized to Sequence A or Sequence B. Two participants in the fasted group experienced AEs prior to receiving the intervention and discontinued the study; these participants were not included in the SAF, PKPS, or BES. The baseline demographics are shown in Table 1. All participants were Chinese, and 69.6% were men. The median age was 32.0 years, the mean (SD) body surface area was 1.73 (0.130) m^2^, the mean (SD) weight was 63.92 (7.219) kg, and the mean (SD) BMI was 22.39 (1.811) kg/m^2^. None of the participants had a history of alcohol consumption or nicotine use.

**TABLE 1:** Baseline demographics.

	Fasted group			Fed group			
	Sequence A (n = 11)	Sequence B (n = 11)	Total (n = 22)	Sequence A (n = 12)	Sequence B (n = 12)	Total (n = 24)	Overall (N = 46)
Sex, n (%)							
Female	3 (27.3)	3 (27.3)	6 (27.3)	4 (33.3)	4 (33.3)	8 (33.3)	14 (30.4)
Male	8 (72.7)	8 (72.7)	16 (72.7)	8 (66.7)	8 (66.7)	16 (66.7)	32 (69.6)
Age, years							
Mean ± SD	33.5 ± 9.06	34.1 ± 8.43	33.8 ± 8.54	27.1 ± 5.93	33.3 ± 6.04	30.2 ± 6.67	31.9 ± 7.75
Median (IQR)	34.0 (24.0–43.0)	38.0 (23.0–41.0)	34.0 (24.0–42.0)	24.5 (22.5–31.5)	33.0 (31.0–37.5)	31.0 (23.5–35.5)	32.0 (24.0–39.0)
Range	22–45	21–44	21–45	22–38	19–42	19–42	19–45
Age category, n (%)							
18–40 years	7 (63.6)	8 (72.7)	15 (68.2)	12 (100.0)	11 (91.7)	23 (95.8)	38 (82.6)
41–64 years	4 (36.4)	3 (27.3)	7 (31.8)	0 (0.0)	1 (8.3)	1 (4.2)	8 (17.4)

In Sequence A, participants received the test product (manufactured in China) and then the reference product (manufactured in Germany); in Sequence B, participants received the reference product and then the test product.

IQR, interquartile range; SD, standard deviation.

### 3.2 PK analysis results

**FIGURE 2:**
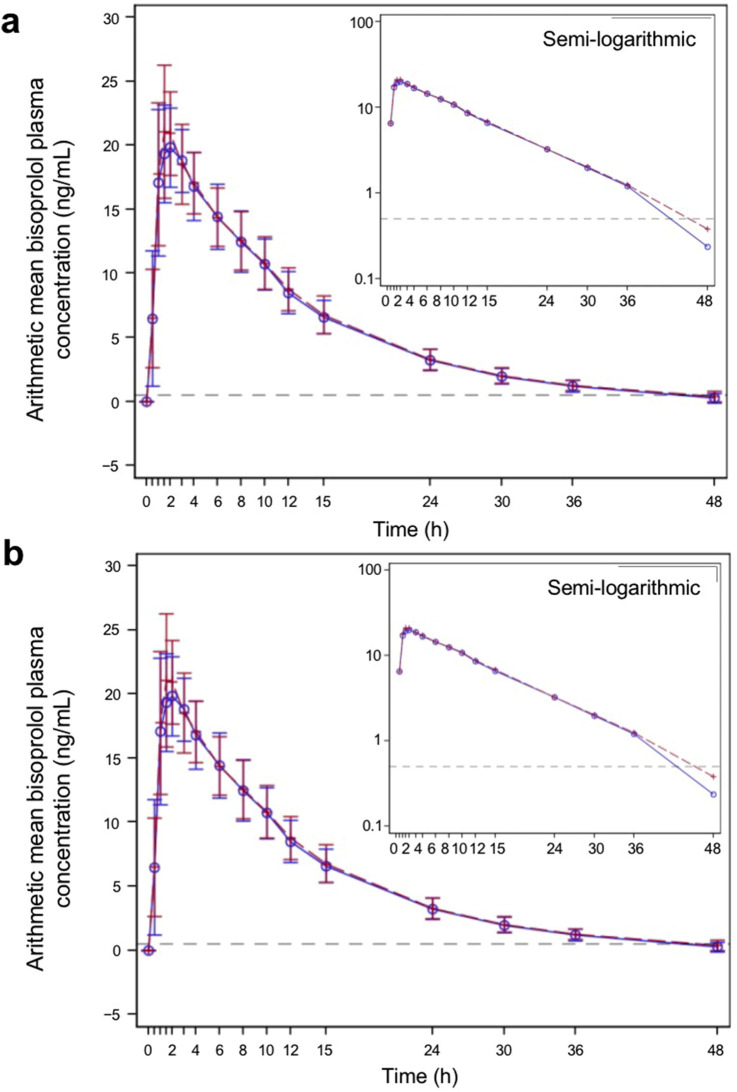
Bisoprolol plasma concentration–time profiles for the test and reference products—pharmacokinetic parameters set **(A)** fasted group and **(B)** fed group. The blue solid line represents participants who received the test product, and the red dashed line represents participants who received the reference product. The test product was bisoprolol 5-mg tablets manufactured in China, and the reference product was bisoprolol 5-mg tablets manufactured in Germany. The gray dashed horizontal line shows the lower level of quantification (0.5 ng/mL). The graphs show linear plots, and the insets are plotted on a semi-logarithmic scale. Individual data points represent the arithmetic mean at each time point, and the error bars show standard deviation.

The PK parameters are summarized in Table 2, and the plasma concentration–time profiles are shown in Figure 2. Following a single dose of the test product and the reference product, the absorption and elimination of bisoprolol were very similar in both the fasted and fed groups. Bisoprolol reached a peak concentration at a median of 1.75 h (test product) and 1.50 h (reference product) in the fasted group and 2.00 h (both test and reference products) in the fed group. In both the fasted and fed groups, both products were eliminated rapidly and plasma levels were below the lower limit of detection after 48 h.

**TABLE 2:** Plasma pharmacokinetic parameters—pharmacokinetic parameters set.

	Fasted group		Fed group	
	Test product n = 20	Reference product n = 20	Test product n = 24	References product n = 24
C_max_, ng/mL				
Mean ± SD	21.4 ± 3.22	22.4 ± 3.73	23.1 ± 4.54	23.1 ± 3.35
Geometric mean (Geometric CV%)	21.2 (15.0)	22.1 (17.0)	22.7 (18.8)	22.8 (15.2)
AUC_0–tlast_, h·ng/mL				
Mean ± SD	257 ± 43.2	264 ± 49.1	251 ± 37.9	261 ± 51.5
Geometric mean (Geometric CV%)	254 (16.5)	260 (19.8)	248 (15.7)	257 (18.8)
AUC_0– ∞ _, h·ng/mL				
Mean ± SD	268 ± 44.0	275 ± 49.5	261 ± 39.1	271 ± 52.0
Geometric mean (Geometric CV%)	265 (16.2)	270 (19.3)	258 (15.6)	266 (18.4)
t_max_, h				
Median (range)	1.750 (1.00–4.00)	1.500 (1.00–3.00)	2.000 (1.00–4.00)	2.000 (1.50–3.00)
CL/F, L/h				
Mean ± SD	19.1 ± 3.01	18.8 ± 3.86	19.6 ± 3.14	19.1 ± 3.31
Geometric mean (Geometric CV%)	18.9 (16.2)	18.5 (19.3)	19.4 (15.6)	18.8 (18.4)
V_z_/F, L				
Mean ± SD	229 ± 33.9	240 ± 49.6	233 ± 28.6	234 ± 33.2
Geometric mean (Geometric CV%)	227 (15.3)	235 (20.5)	231 (12.8)	232 (15.8)
t_1/2_, h				
Median (range)	8.169 (6.86–11.67)	8.665 (6.84–12.17)	8.090 (6.73–10.96)	8.357 (7.26–11.10)

AUC_0–tlast_, area under the plasma concentration–time curve from time 0 to the time of the last measurable concentration; AUC_0–__ ∞ _, area under the plasma concentration–time curve from time 0 to infinity; CL/F, apparent plasma clearance of the drug following extravascular administration; C_max_, maximum observed concentration; CV, coefficient of variation; SD, standard deviation; t_1/2_, elimination half-life; t_max_, time of the maximum observed concentration; V_z_/F, apparent volume of distribution during the terminal phase following extravascular administration.

**FIGURE 3:**
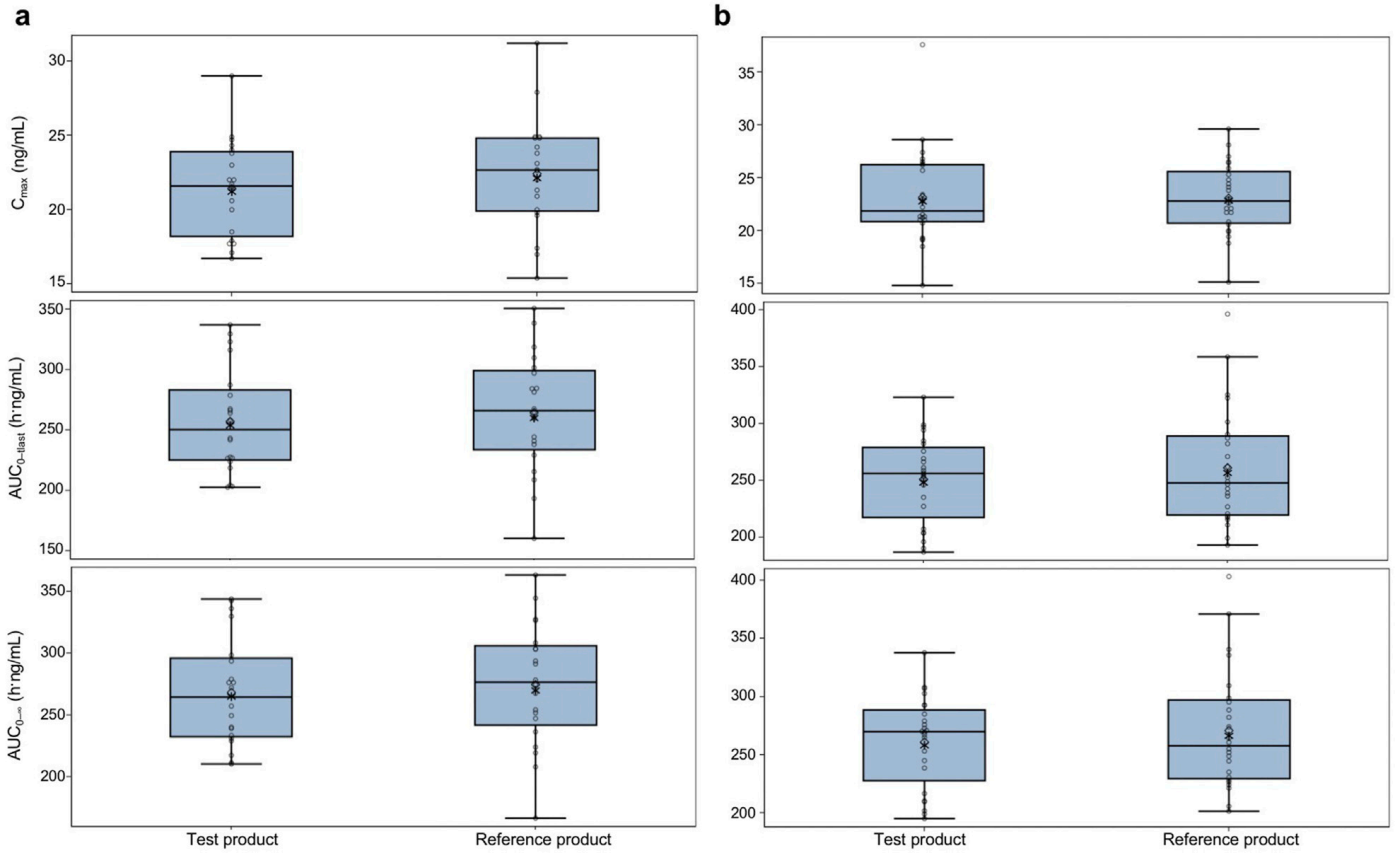
Pharmacokinetic parameters for the test and reference products in fasted and fed healthy participants—pharmacokinetic parameters set **(A)** fasted group and **(B)** fed group. The test product was bisoprolol 5-mg tablets manufactured in China, and the reference product was bisoprolol 5-mg tablets manufactured in Germany. The solid horizontal line represents the median value, the diamonds are the arithmetic mean, and the asterisks in the box are the geometric mean values. The upper and lower edges of the box are the 1^st^ and 3^rd^ quartiles, respectively, and the whiskers are the range, excluding outliers. Outliers were considered values that were outside 1.5 × the interquartile range. AUC_0–tlast_, area under the plasma concentration–time curve from time 0 to the time of the last measurable concentration; AUC_0– ∞ _, area under the plasma concentration–time curve from time 0 to infinity; C_max_, maximum observed concentration.

In the fasted group, the geometric mean (geometric CV percentage) C_max_ was 21.2 (15.0) ng/mL for the test product and 22.1 (17.0) ng/mL for the reference product. In the fed group, the geometric mean (geometric CV percentage) C_max_ was 22.7 (18.8) ng/mL for the test product and 22.8 (15.2) ng/mL for the reference product. Both AUC_0–tlast_ and AUC_0– ∞_ of bisoprolol were similar for the test and reference products under both fasted and fed conditions. The mean t_1/2_, V_z_/F, and CL/F were also similar for both products. Boxplots of C_max_, AUC_0–tlast_, and AUC_0– ∞_ in both the fasted and fed groups are shown in Figure 3. PK parameters were similar between the test and reference products and between both the fasted and fed groups.

### 3.3 Bioequivalence

The bioequivalence analysis results are shown in Table 3. Among the fasted group, the geometric LS mean ratio (90% CI) for the test product/reference product was 0.9565 (0.9006–1.0158) ng/mL for C_max_, 0.9761 (0.9370–1.0168) h·ng/mL for AUC_0–tlast_, and 0.9807 (0.9429–1.0200) h·ng/mL for AUC_0– ∞ _. As all CIs were within the range of 0.80–1.25, this met the pre-defined criteria for bioequivalence. In the fed group, the geometric LS mean ratio (90% CI) for the test product/reference product was 0.9966 (0.9289–1.0691) ng/mL for C_max_, 0.9672 (0.9220–1.0145) h·ng/mL for AUC_0–tlast_, and 0.9693 (0.9253–1.0155) h·ng/mL for AUC_0– ∞ _. This also met the pre-defined criteria for bioequivalence.

**TABLE 3:** Bioequivalence analysis—bioequivalence set.

	Geometric LS mean (95% CI)	Ratio (test product/reference product)	90% CI of ratio	Intra-CV (%)
Fasted (n = 20)				
C_max_, ng/mL				
Test product	21.17 (19.63–22.83)	0.9565	0.9006–1.0158	11.0
Reference product	22.13 (20.53–23.86)			
AUC_0–tlast_, h·ng/mL				
Test product	254.70 (233.93–277.33)	0.9761	0.9370–1.0168	7.4
Reference product	260.95 (239.66–284.12)			
AUC_0– ∞ _, h·ng/mL				
Test product	265.98 (244.82–288.97)	0.9807	0.9429–1.0200	7.1
Reference product	271.21 (249.63–294.65)			
Fed (n = 24)				
C_max_, ng/mL				
Test product	22.75 (21.20–24.41)	0.9966	0.9289–1.0691	14.3
Reference product	22.83 (21.28–24.49)			
AUC_0–tlast_, h·ng/mL				
Test product	248.13 (231.38–266.11)	0.9672	0.9220–1.0145	9.7
Reference product	256.56 (239.23–275.14)			
AUC_0– ∞ _, h·ng/mL				
Test product	258.20 (240.94–276.70)	0.9693	0.9253–1.0155	9.4
Reference product	266.37 (248.56–285.45)			

AUC_0–tlast_, area under the plasma concentration–time curve from time 0 to the time of the last measurable concentration; AUC_0– ∞ _, area under the plasma concentration–time curve from time 0 to infinity; CI, confidence interval; C_max_, maximum observed concentration; intra-CV, intra-participant coefficient of variation; LS, least squares.

### 3.4 Dissolution results

**FIGURE 4:**
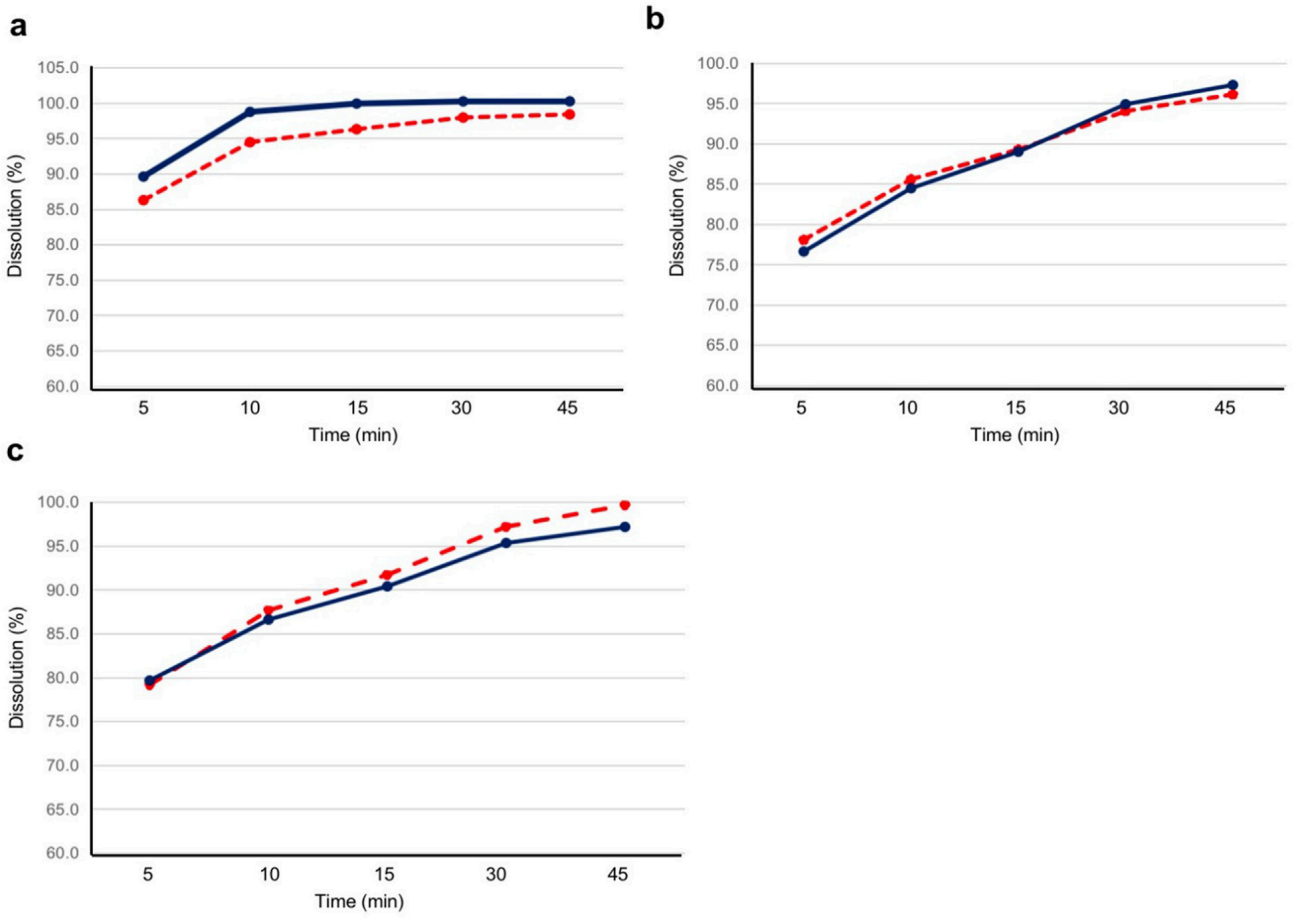
Dissolution profile for the test and reference products at pH 1.2, 4.5, and 6.8. Twelve tablets for each product were evaluated for dissolution at **(A)** pH 1.2, **(B)** pH 4.5, and **(C)** pH 6.8. The blue solid line represents the test product, and the red dashed line represents the reference product. The test product was bisoprolol 5-mg tablets manufactured in China, and the reference product was bisoprolol 5-mg tablets manufactured in Germany.

The dissolution assay results of the test and reference products are shown in Figure 4. At all pH conditions (pH 1.2, pH 4.5, and pH 6.8), the mean dissolution rates for both the test and reference products were higher than 85% at 15 min.

### 3.5 Safety

The TEAE data are tabulated in Table 4. A higher frequency of TEAEs occurred following the administration of the reference product (10/44 [22.7%]) than after the test product (4/44 [9.1%]). Three participants in the fed group experienced TEAEs considered to be related to the study treatment (two product-related first-degree atrioventricular block events and one product-related diastolic blood pressure decrease). Both first-degree atrioventricular block events were experienced by one participant, once with the test and once with the reference product, and the diastolic blood pressure decrease event occurred with the reference product. All product-related TEAEs were mild in severity and resolved. None of the participants discontinued because of TEAEs, and no serious TEAEs or deaths occurred.

**TABLE 4:** Treatment-emergent adverse events—safety analysis set.

	Fasted group			Fed group			Overall		
	Test product (n = 20)	Reference product (n = 20)	Total (n = 20)	Test product (n = 24)	Reference product (n = 24)	Total (n = 24)	Test product (n = 44)	Reference product (n = 44)	Total (N = 44)
Any TEAE	0 (0.0)	3 (15.0)	3 (15.0)	4 (16.7)	7 (29.2)	10 (41.7)	4 (9.1)	10 (22.7)	13 (29.5)
Any product-related TEAE	0 (0.0)	0 (0.0)	0 (0.0)	1 (4.2)	2 (8.3)	2 (8.3)	1 (2.3)	2 (4.5)	2 (4.5)
Primary system organ class preferred term									
Investigations	0 (0.0)	3 (15.0)	3 (15.0)	3 (12.5)	6 (25.0)	9 (37.5)	3 (6.8)	9 (20.5)	12 (27.3)
Blood triglyceride increase	0 (0.0)	0 (0.0)	0 (0.0)	1 (4.2)	5 (20.8)	6 (25.0)	1 (2.3)	5 (11.4)	6 (13.6)
Blood uric acid increase	0 (0.0)	3 (15.0)	3 (15.0)	1 (4.2)	0 (0.0)	1 (4.2)	1 (2.3)	3 (6.8)	4 (9.1)
Diastolic blood pressure decreased	0 (0.0)	0 (0.0)	0 (0.0)	0 (0.0)	1 (4.2)	1 (4.2)	0 (0.0)	1 (2.3)	1 (2.3)
Urinary occult blood positive	0 (0.0)	0 (0.0)	0 (0.0)	1 (4.2)	0 (0.0)	1 (4.2)	1 (2.3)	0 (0.0)	1 (2.3)
Cardiac disorders	0 (0.0)	0 (0.0)	0 (0.0)	1 (4.2)	1 (4.2)	1 (4.2)	1 (2.3)	1 (2.3)	1 (2.3)^a^
First-degree atrioventricular block	0 (0.0)	0 (0.0)	0 (0.0)	1 (4.2)	1 (4.2)	1 (4.2)	1 (2.3)	1 (2.3)	1 (2.3)

Adverse events were coded using Medical Dictionary for Regulatory Activities Version 25.0. Data are shown as n (%).

^a^ This participant experienced an event (two events total) with both the test and reference products.

TEAE, treatment-emergent adverse event.

## 4 Discussion

This study demonstrated and compared the bioequivalence of bisoprolol 5-mg tablets manufactured in China to tablets manufactured in Germany in healthy Chinese adults under both fasted and fed conditions. Moreover, both tablets were well-tolerated, and no unexpected safety concerns were encountered. Importantly, this study provides evidence to meet regulatory filing requirements, thereby supporting Merck’s improved manufacturing capabilities for bisoprolol and access to bisoprolol for Chinese patients.

The bisoprolol PK results were generally consistent with those of previous reports in both Chinese participants (Hu et al., 2018; Wang et al., 2009; Zhou et al., 2007) and participants of other ethnicities (Tjandrawinata et al., 2012; Leopold et al., 1986). A previous study reported the PK profile of bisoprolol in Chinese participants who received both bisoprolol and amlodipine (Hu et al., 2018). The lack of PK drug–drug interactions between bisoprolol and amlodipine allows comparison between the bisoprolol PK profile in the previous study and the current study. Among participants who received both bisoprolol and amlodipine, the bisoprolol C_max_ geometric means were approximately 26 ng/mL in the fasted group and 21 ng/mL in the fed group, AUC_0–tlast_ geometric means were approximately 294 h·ng/mL in the fasted group and 270 h·ng/mL in the fed group, and AUC_0– ∞_ geometric means were approximately 306 h·ng/mL in the fasted group and 285 h·ng/mL in the fed group (Hu et al., 2018). These findings are similar to those in the current study, where the C_max_ geometric means were approximately 22 ng/mL in both fasted and fed groups, AUC_0–tlast_ geometric means were approximately 260 h·ng/mL in the fasted group and 255 h·ng/mL in the fed group, and AUC_0– ∞_ geometric means were approximately 270 h·ng/mL in the fasted group and 265 h·ng/mL in the fed group. Thus, the bisoprolol PK profile reported in this current study is consistent with previous reports of bisoprolol in Chinese participants.

As the geometric LS mean ratios for C_max_, AUC_0–tlast_, and AUC_0– ∞_ were within 0.95–1.05, and the 90% CI of the ratios were within 0.80–1.25, this study confirmed the bioequivalence of bisoprolol 5-mg tablets manufactured in China and those manufactured in Germany. All other PK parameters evaluated were also similar between the two products. Moreover, the dissolution profiles of the test and reference products showed similar results in all three pH conditions, further supporting the comparability of the two products. This study also found that the tablets manufactured in China and Germany exhibited bioequivalence under both fasted and fed conditions, with similar PK profiles for bisoprolol under each condition, as observed previously (Tjandrawinata et al., 2012; Hu et al., 2018; Leopold et al., 1986).

The safety results showed that the tablets manufactured both in China and in Germany were generally safe and well-tolerated. All product-related TEAEs (first-degree atrioventricular block and increase in diastolic blood pressure) were mild and resolved. Additionally, most reported TEAEs were events that were known for the product (including increase in blood triglyceride levels) (Merck, 2014). Both the test and reference products had a similar frequency of TEAEs, and there were no new or unexpected safety concerns.

This study had some limitations: Concor^®^ is approved at dosages of 5 mg and 10 mg. Single-dose BE studies commonly use the highest dose strength, unless participant safety is a concern. In this study, the 5-mg dose was selected due to its clinical relevance as the most commonly prescribed starting dose and to minimize potential safety risks to healthy volunteers. Regulatory guidelines permit the use of a lower dose strength when justified by safety considerations (U.S. Food and Drug Administration, 2021). The 5-mg dose ensured sufficient bioanalytical sensitivity while maintaining participant safety. Due to the limitations of the sample size and single-dose administration, it was difficult to conduct a comprehensive assessment of the safety of the two drugs, and potential long-term safety concerns need to be further perfected. No formal statistical comparisons were conducted regarding the secondary endpoints. Additionally, although no differences in PK parameters were identified between the fasted and fed groups, this finding should be interpreted with caution as no formal comparisons were conducted.

In conclusion, this study found that bisoprolol 5-mg tablets manufactured in China exhibited bioequivalence comparable to that of tablets manufactured in Germany in healthy Chinese adults, and that the tablets were both well-tolerated and had comparable safety profiles.

## Data Availability

The raw data supporting the conclusions of this article will be made available by the authors, without undue reservation.
